# Biomarker relationships with small bowel histopathology among malnourished children with environmental enteric dysfunction in a multicountry cohort study

**DOI:** 10.1016/j.ajcnut.2024.02.029

**Published:** 2024-09-17

**Authors:** Mustafa Mahfuz, David Coomes, Marwa Abdalla, Monica Mweetwa, Kelley VanBuskirk, Najeeha T Iqbal, S Asad Ali, Kanta Chandwe, Subhasish Das, Paul Kelly, Nurmohammad Shaikh, Phillip I Tarr, Donna M Denno, EEDBI Consortium, Kumail Ahmed, Sheraz Ahmed, Tahmeed Ahmed, Md. Ashraful Alam, Beatrice Amadi, S.M. Khodeza Nahar Begum, Ellen Besa, Shah Mohammad Fahim, Md. Amran Gazi, Carol A. Gilchrist, Rashidul Haque, Md. Mehedi Hasan, Md. Shabab Hossain, Aneeta Hotwani, Shahneel Hussain, Junaid Iqbal, Sadaf Jakhro, Furqan Kabir, Ta-Chiang Liu, Barbara J. Mann, Ramendra Nath Mazumder, Waheeda Memon, Christopher A Moskaluk, Abdul Khalique Qureshi, Shyam S Ragahavan, Masudur Rahman, Najeeb Rahman, Kamran Sadiq, Shafiqul Alam Sarker, Peter B Sullivan, Guillermo J. Tearney, Fayaz Umrani, Omer H. Yilmaz, Kanekwa Zyambo

**Affiliations:** 10 Department of Paediatrics and Child Health, Aga Khan University, Karachi, Pakistan; 11 International Centre for Diarrhoeal Disease Research, Bangladesh, Dhaka, Bangladesh; 12 Tropical Gastroenterology and Nutrition Group, University of Zambia School of Medicine, Lusaka, Zambia; 13 Department of Pathology, Bangladesh Specialized Hospital, Dhaka, Bangladesh; 14 Tropical Gastroenterology & Nutrition group, University of Zambia School of Medicine, Lusaka, Zambia; 15 Department of Medicine, University of Virginia, Charlottesville, Virginia, USA; 16 Infectious Diseases Division, International Centre for Diarrhoeal Disease Research, Bangladesh, Dhaka, Bangladesh; 17 Department of Biological and Biomedical Sciences, Aga Khan University, Karachi, Pakistan; 18 Department of Pathology and Immunology, Washington University, St. Louis, MO, USA; 19 Department of Pathology, University of Virginia School of Medicine, Charlottesville, VA, USA; 20 Department of Gastroenterology, Sheikh Russel National Gastroliver Institute and Hospital, Dhaka, Bangladesh; 21 Department of Paediatrics, Children's Hospital, University of Oxford, Oxford, UK; 22 Department of Pathology, Harvard Medical School, Boston, MA, USA; 23 Department of Pathology, Massachusetts General Hospital, Boston, MA, USA; 1 Nutrition Research Division, International Centre for Diarrhoeal Disease Research, Bangladesh, Dhaka, Bangladesh; 2 Department of Epidemiology, University of Washington School of Public Health, Seattle, WA, United States; 3 Tropical Gastroenterology & Nutrition Group, University of Zambia School of Medicine, Lusaka, Zambia; 4 Department of Global Health, University of Washington School of Public Health, Seattle, WA, United States; 5 Department of Paediatrics and Child Health, Aga Khan University, Karachi, Pakistan; 6 Blizard Institute, Barts & The London School of Medicine, Queen Mary University of London, London, United Kingdom; 7 Department of Pediatrics, Washington University School of Medicine, St. Louis, MO, United States; 8 Department of Pediatrics, University of Washington School of Medicine, Seattle, WA, United States

**Keywords:** EED, biomarkers, intestinal inflammation, intestinal permeability, children, low-resource settings

## Abstract

**Background:**

Validated biomarkers could catalyze environmental enteric dysfunction (EED) research.

**Objectives:**

Leveraging an EED histology scoring system, this multicountry analysis examined biomarker associations with duodenal histology features among children with EED. We also examined differences in 2-h compared with 1-h urine collections in the lactulose rhamnose (LR) dual sugar test.

**Methods:**

Three cohorts of undernourished children unresponsive to nutrition intervention underwent esophagogastroduodenoscopy and duodenal biopsies. Histopathology scores were compared to fecal calprotectin (CAL), myeloperoxidase (MPO), neopterin (NEO), and urinary LR ratio and lactulose percentage recovery. Log-transformed biomarkers were used in linear regressions adjusted for age, center, and sample collection–biopsy time interval in multivariable models.

**Results:**

Data on >1 biomarker were available for 120 Bangladeshi (CAL, MPO, NEO, and LR), 63 Pakistani (MPO, NEO, and LR), and 63 Zambian children (CAL). Median age at endoscopy was similar (19 mo) across centers. Median sample collection prior to endoscopy was consistent with each center’s study design: 2 wk in Bangladesh (urine and stool) and Zambia (stool), and 6 (urine) and 11 (stool) mo in Pakistan. In multivariable models, intraepithelial lymphocytes were associated with CAL (exponentiated [exp.] coefficient: 1.19; 95% confidence interval [CI]: 1, 1.41), intramucosal Brunner’s glands with MPO (exp. coefficient: 1.33; 95% CI: 1.05, 1.69) and NEO (exp. coefficient: 1.37; 95% CI: 1.1, 1.7), and chronic inflammation with NEO (exp. coefficient: 1.61; 95% CI: 1.17, 2.17). Intraepithelial lymphocytes were associated with lactulose % recovery (exp. coefficient: 1.22; 95% CI: 1.05, 1.41). LR recovery was substantially lower in 1-h collections than in 2-h collections.

**Conclusions:**

Four commonly used markers of enteric dysfunction were associated with specific histologic features. One-hour urine collection may be insufficient to reflect small bowel permeability in LR testing. While acknowledging the challenges with obtaining relevant tissue, these findings form the basis for further EED biomarker validation research.

## Introduction

Environmental enteric dysfunction (EED) is an asymptomatic acquired disorder of elusive etiology that is highly prevalent among children living in low-resource settings with suboptimum sanitary conditions [1,2]. EED is characterized by structural and functional changes in the small intestinal mucosa causing reduced absorption of nutrients, increased gut permeability, and local and systemic inflammation [2]. EED has been associated with childhood growth faltering [3]. Most EED studies have relied on stool or plasma biomarkers or dual sugar permeability tests, but data are inconsistent across studies and geographical locations. For example, one longitudinal study of Bangladeshi children observed an association between stool myeloperoxidase (MPO) concentrations with linear growth [4]; however, another study in Zimbabwe observed no such association [5]. Moreover, biomarkers have not been validated with small intestinal histology because endoscopy in children in EED endemic settings has been uncommon due to cost, expertise required, risk considerations, and lack of guidelines in low- and middle-income countries on indications for the procedure.

However, recent advances in histologic characterization and the development and validation of a scoring system by the Environmental Enteric Dysfunction Biopsy Initiative (EEDBI) Consortium [6,7] now permit the interrogation of relationships between predictors, outcomes, and other factors of interest with tissue histology. Three independent studies comprising the EEDBI Consortium were conducted in Bangladesh, Pakistan, and Zambia. Endoscopies were performed and duodenal mucosal biopsies were analyzed to study EED in undernourished young children who did not respond to nutritional intervention and who had an otherwise negative medical workup [6,7]. These studies also performed assays to test an array of biomarkers from less-invasively collected specimens, including stool and urine. The EEDBI Consortium, therefore, offers a unique opportunity to assess the relationship of biomarkers collected across multiple geographic regions with histopathology of the disease tissue target—the small bowel. Specifically, we aimed to identify whether 4 biomarkers, including 3 common fecal markers of intestinal inflammation (MPO, calprotectin [CAL], and neopterin [NEO]) and a dual sugar permeability (lactulose rhamnose [LR]) test, are associated with individual histologic features of EED and an EED summary index score. Additionally, for the LR test, compared to the traditional practice of 3- to 5-h postingestion urine collection, a recent study suggested that a 60-min collection interval may be sufficient as an assessment of small intestinal permeability when the urinary analytes are measured using high-performance liquid chromatography-mass spectrometry (HPLC-MS), which is highly sensitive [8]. Therefore, we sought to compare urinary sugar results measured by HPLC-MS from 1-h only to 2-h urine collections.

## Methods

### Subjects and methods

Study participants included children enrolled in Biomarkers of Environmental Enteropathy in Children (BEECH), Bangladesh Environmental Enteric Dysfunction (BEED), and Study of Environmental Enteropathy and Malnutrition (SEEM) studies conducted in Zambia, Bangladesh, and Pakistan, respectively, who underwent esophagogastroduodenoscopy and small bowel biopsies and for whom biomarker data were available. Detailed study methodologies, including recruitment and sampling timeframes, enrollment procedures, eligibility criteria, and nutritional management as well as characteristics of study participants are described elsewhere [6,[9], [10], [11]].

In brief, the BEECH study was conducted in urban Lusaka where children <18 mo of age with wasting (weight-for-length Z score <−2) or stunting (length-for-age Z score [LAZ] <−2) insufficiently responsive to 3–4 mo of nutritional intervention underwent esophagogastroduodenoscopy and biopsy. The BEED study was conducted in urban Dhaka where children aged 12–18 mo were enrolled in 2 groups—stunted and “at risk for stunting” (LAZ < −1 to ≥ −2). Those without improvement in their linear growth category after 3 mo of nutritional intervention underwent esophagogastroduodenoscopy and biopsy. In the SEEM study, 9-mo-olds with wasting from rural Sindh province were provided nutritional intervention for 2 mo; those with insufficient response underwent esophagogastroduodenoscopy and biopsy. Endoscopy exclusion criteria across the 3 sites included a recent history of diarrhea, laboratory findings consistent with a risk factor for an underlying bleeding disorder, a medically identifiable etiology of refractory undernutrition, including tissue transglutaminase immunoglobulin A levels suggestive of celiac disease. Biopsy samples were collected across studies between November 2016 and August 2019. Further details are provided in a companion paper in this series [6].

### Biomarkers

Stools were collected for biomarkers across all 3 studies although timing differed; BEED and BEECH protocols called for sample collection after nutritional intervention and before endoscopy, whereas the SEEM protocol specified collection at ages 6 and 9 mo. Urinary dual sugar (LR) permeability testing was conducted by the SEEM and BEED studies. We analyzed markers of intestinal dysfunction that were assessed at more than one of the study centers. These included urinary dual sugars as a purported measure of gut permeability and 3 fecal markers thought to be measures of intestinal inflammation—MPO, CAL, and NEO. We included biomarker data from samples collected closest to the time of biopsy if more than one sample was obtained.

### Laboratory methods: fecal biomarkers

Frozen stools were thawed on ice and enzyme-linked immunosorbent assays were performed according to manufacturers’ instructions. The kits used are listed in Table 1. Assays were performed in duplicate. Biomarker concentrations were extrapolated from each assay’s standard curve. Values above or below the standard curve were typically rerun at adjusted dilutions. However, if a sample could not be rerun, values above and below the curves were entered into analysis as the maximum value on the curve or the midpoint between the lower limit of detection and zero, respectively. Blanks were subtracted from measured reads.

**TABLE 1:** Fecal biomarker kits, by the center

	BEECH	BEED	SEEM
Calprotectin	Immundiagnostik (IDK) Calprotectin (stool) ELISA (K6927)	Buhlmann (catalog #EK-CAL)	
Myeloperoxidase	Epitope Diagnostics Inc, ELISA KT-880^1^	Alpco (K-6630)	Alpco (K-6630)
Neopterin		Genway (40-371-25012)	Genway (40-371-25012)

Abbreviations: BEECH, Biomarkers of Environmental Enteropathy in Children; BEED, Bangladesh Environmental Enteric Dysfunction; SEEM, Study of Environmental Enteropathy and Malnutrition.

1 Results from this kit substantially differed from the kit used by BEED and SEEM; hence, BEECH myeloperoxidase data were excluded from further analysis.

### Laboratory testing: LR dual sugar permeability testing

BEED and SEEM used similar procedures for LR testing based on a recently published protocol [8]. Participants fasted 1 h prior to sugar solution ingestion. Before fasting and 30 min after dosing, participants were encouraged to breastfeed or drink water (SEEM) or cow’s milk (BEED). The sugar solution containing 200 mg L-rhamnose (TCI Chemicals, product R0013) and 1000 mg lactulose (Sigma-Aldrich, product 61360) in 10 mL sterile water was administered over 5 min. Collection bags were placed to collect predose urine from subsamples of participants to assess for possible ambient exposure to the sugars (Supplemental Table 1) after 1 h of fasting, and postdose urine 20 min (SEEM) and 30 min (BEED) after sugar solution ingestion for 2 60-min intervals. The bags were changed between the 2 points. SEEM split the last interval into 2 30-min collections, although only 3 children produced specimens at both time points; data from these 2 samplings were pooled for this analysis. The postdose 1-h urine was extended by 30 min (90 min after dose) if the child did not void in the first hour. Urine samples were aliquoted and placed into cooler bags, then transferred to each central lab for storage at −80°C prior to shipment to the Mayo Clinic for quantification of sugar concentrations by HPLC-MS per a previously published protocol [8].

### Duodenal biopsy histology scoring

The histology scoring system and procedures are described in detail in a companion paper in this series [7]. In brief, endoscopic mucosal pinch biopsies from the second or third duodenal segment were sectioned and stained with hematoxylin and eosin. Slide images uploaded to a telepathology platform were semiquantitatively scored across 8 histologic parameters by 2 or 3 Consortium gastrointestinal pathologists (each parameter and scores are defined in Supplemental Table 2). Slide consensus scores for each parameter were averaged across pathologist readings. Finally, a global index score—the total score percent-5 (TSP-5)—developed to identify EED and assign EED histopathologic severity, was calculated from the 5 histology parameters most informative in differentiating EED from a reference group of American children without a clinical or histopathologic diagnosis of gastrointestinal disorders: villus blunting, intraepithelial lymphocytes (IELs), goblet and Paneth cell depletion, and intramucosal Brunner’s glands. If more than one of these 5 parameters were not scorable because of technical issues, the TSP-5 was also considered nonscorable. When more than one slide image for an individual was available, average scores across slides were used.

### Statistical methods

Summary statistics of fecal biomarker concentrations, the LR ratio (L:R), and lactulose percentage (L%) recovery were calculated; we excluded one clinically implausible measurement (MPO).

The following formula was used to calculate cumulative L% recovery over the 2-h collection period:

$$\frac{\begin{array}{c} \left(L\;concentration\;1st\;hour\;\left(\mu g/mL\right)\times urine\;volume\;1st\;hour\;\left(mL\right)\right)+\\ \left(L\;concentration\;2nd\;hour\;\left(\mu g/mL\right)\times urine\;volume\;2nd\;hour\;\left(mL\right)\right) \end{array}}{Initial\;L\;dose\;\left(\mu g\right)}\times 100$$

We used the following formula to calculate the overall L:R across both time points:

$$\frac{L\;\%\;{recovery\;1}^{st}\;hour+L\;\%\;recovery\;2^{nd}\;hour}{R\;\%\;recovery\;1^{st}\;hour+R\;\%\;{recovery\;2}^{nd}\;hour}$$

To test the association of CAL, MPO, NEO, L:R, and L% recovery with the TSP-5 or individual histopathologic parameters, we performed linear regression including biomarker measurements as outcomes and histologic scores as predictors. After examining regression residuals for normality, biomarker values were log-transformed before being included in the final models.

Nonscorable responses for histologic variables were treated as missing data. Associations from univariate models with a *P* value < 0.1 were further explored in multivariable models that adjusted for the potential confounding effects of age (mo), study center (BEECH, BEED, and SEEM), and time between sample collection and biopsy (d). Statistical significance in this study was defined as *P* < 0.05, and all reported *P* values are 2-sided. Power calculations are provided in the Appendix A. Analyses were performed with R (version 4.1.1, R Foundation) and Stata (version 15).

### Ethical approvals

Signed informed consent from legal guardians was obtained, and all study centers obtained approval for these studies from their respective review boards. Details on these procedures as well as information regarding previously published single-center data relevant to this multicenter biomarkers analysis can be found elsewhere in this supplemental issue [6].

## Results

**FIGURE 1:**
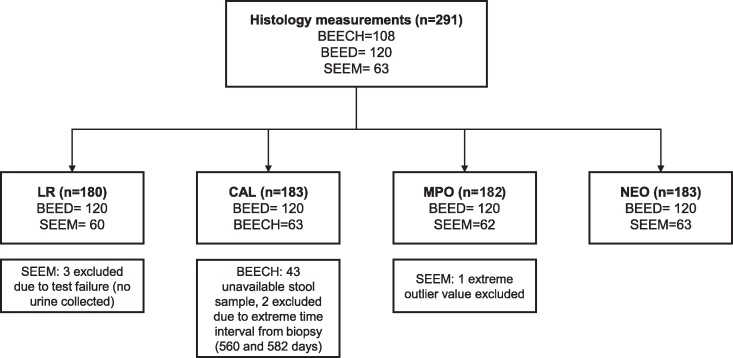
Total number of participants per biomarker analysis per center. BEECH, Biomarkers of Environmental Enteropathy in Children; BEED, Bangladesh Environmental Enteric Dysfunction; CAL, calprotectin; LR, lactulose rhamnose; MPO, myeloperoxidase; NEO, neopterin; SEEM, Study of Environmental Enteropathy and Malnutrition.

Two hundred forty-six children across the 3 centers who underwent endoscopy had data for at least one biomarker that was tested in common with another center (Figure 1). The median age at the time of endoscopy was 19 mo across the centers (Table 2). SEEM had a greater proportion of males enrolled (70%) compared to BEECH (49%) and BEED (42%). The median LAZ in the BEECH and SEEM cohorts (−3.3 and −3.2, respectively) was lower than for BEED (−2.1). The SEEM cohort was most affected by wasting and underweight. Table 2 also describes the histology scores of duodenal biopsy slide images. Median TSP-5 scores were highest among the BEED cohort (59%), followed by BEECH (56%) and SEEM (45%). Goblet and Paneth cells were most depleted in the BEED biopsies whereas intraepithelial lymphocytosis was most prominent in the SEEM and BEED biopsies and villus architecture was most disrupted among the BEECH biopsies.

**TABLE 2:** Descriptive statistics of the study participants with at least one biomarker result

Measurement	BEECH *n* = 63	BEED *n* = 120	SEEM *n* = 63	Combined *n* = 246
	median (IQR)			
Age (mo), at endoscopy	18.7 (15.1, 21.3)	19.1 (17.3, 20.7)	20.4 (15.1, 21.9)	19.1 (16.8, 21.3)
Age (mo), at stool collection	17.4 (12.7, 20.7)	18.5 (16.9, 20.3)	9.2 (9.1, 9.3)	16.9 (9.4, 19.9)
Age (mo), at urine collection	-	18.5 (16.9, 20.3)	13.3 (12.1, 15.2)	17.1 (15.3, 19.7)
Female (%)	32 (50.8)	70 (58.3)	19 (30.2)	121 (49.2)
LAZ^1^	−3.3 (−4.0, −3.0)	−2.1 (−2.8, −1.5)	−3.2 (−3.7, −2.4)	−2.8 (−3.4, −2.0)
WLZ^1^	−0.7 (−1.3, −0.2)	−1.0 (−1.4, −0.4)	−2.2 (−2.8, −1.8)	−1.1 (−1.9, −0.5)
WAZ^1^	−2.3 (−2.7, −1.8)	−1.7 (−2.3, −1.2)	−3.1 (−3.6, −2.6)	−2.2 (−2.9, −1.6)
Histology parameter (range of possible scores)	median (IQR)/% not scorable			
Total score percent-5 (0–100%)	55.6 (49.1, 61.1)/6.3%	58.6 (47.2, 66.7)/33.3%	44.5 (36.7, 52.4)/0%	52.8 (44.4, 61.1)/17.9%
Goblet cell depletion (0–4)	1.5 (1.0, 2.0)/3.2%	2.0 (1.4, 2.5)/0%	1.0 (0.8, 1.3)/0%	1.5 (1.0, 2.0)/0.8%
Intraepithelial lymphocytes (0–4)	0.9 (0.5, 1.5)/1.6%	1.5 (1.0, 2.0)/0.8%	1.7 (1.2, 2.5)/0%	1.5 (1.0, 2.0)/0.8%
Intramucosal Brunner’s glands (0–3)	0.0 (0.0, 0.3)/1.6%	0.0 (0.0, 0.5)/3.3%	0.2 (0.0, 1.0)/0%	0.0 (0.0, 0.5)/2.0%
Paneth cell depletion (0–3)	2.5 (1.5, 3.0)/15.9%	3.0 (1.9, 3.0)/46.7%	0.7 (0.5, 1.0)/1.6%	1.6 (0.8, 3.0)/27.2%
Villus architecture (0–4)	2.5 (2.0, 3.5)/9.5%	2.0 (1.0, 3.0)/39.2%	2.1 (1.3, 3.0)/9.5%	2.0 (1.5, 3.2)/24.0%
Chronic inflammation (0–3)	1.5 (1.2, 2.0)/3.2%	1.5 (1.0, 1.5)/1.7%	1.3 (1.0, 1.6)/0%	1.5 (1.0, 1.7)/1.6%
Enterocyte injury (0–3)	0.3 (0.0, 0.5)/1.6%	0.3 (0.0, 0.5)/0%	0.3 (0.2, 0.5)/0%	0.3 (0.0, 0.5)/0.4%
Epithelial detachment (0–4)	1.0 (0.7, 1.1)/0%	1.0 (0.5, 1.0)/0%	1.0 (0.8, 1.2)/0%	1.0 (0.7, 1.2)/0%

Abbreviations: BEECH, Biomarkers of Environmental Enteropathy in Children; BEED, Bangladesh Environmental Enteric Dysfunction; LAZ, length-for-age Z score; SEEM, Study of Environmental Enteropathy and Malnutrition; WAZ, weight-for-age Z score; WLZ, weight-for-length Z score.

1 Measurement at the time closest to biopsy. Recumbent length is measured for children <2 y of age, while standing height is measured for those ≥ 2y of age. 4 children in the BEECH analysis were 2y of age at the anthropometric measurement closest to the time of biopsy.

Table 3 presents a summary of biomarker assay results. Stool collection time intervals from biopsy were consistent with study protocols: the median for BEECH and BEED was on the day of and 2 wk prior to the procedure, respectively, and 11 mo prior for SEEM (at median 9 mo of age [Table 2]). LR testing was conducted within a median of 2 and 30 wk prior to endoscopy for BEED and SEEM, respectively.

**TABLE 3:** Summary of calprotectin, myeloperoxidase, neopterin, and lactulose rhamnose results∗

Measurement	BEECH	BEED	SEEM	Combined
Biomarker sample biopsy time interval^1^, d				
CAL	0 (0, 124.5)	14 (10, 24)	—	13 (3, 25)
MPO	—	14 (10, 24)	343.0 (178.3, 387.3)	24 (13, 180.8)
NEO	—	14 (10, 24)	342.0 (179.5, 386.5)	24 (13, 188.5)
LR	—	14 (9, 22)	209 (40, 275)	20 (12, 64)
Fecal biomarker concentrations				
*n*^2^	63	120	63	246
CAL (μg/g)	211 (127.2, 386.6)	374 (195.0, 600.8)	—	291 (164.2, 551.8)
MPO (ng/mL)	—	1847 (748.0, 4418.9)	3108 (1551.1, 6365.5)	2495 (829.8, 4892.8)
NEO (μg/mL)	—	1117 (486.2, 2486.5)	1950(825.0, 2837.5)	1315 (557.5, 2650.0)
Urine collection for LR, *n* (%)				
Void only first hour	—	4 (3.3)	13 (21.7)	17 (9.4)
Void only second hour	—	16 (13.3)	8 (13.3)	24 (13.3)
Voided both hours	—	100 (83.3)	39 (65.0)	139 (77.2)
Urinary LR				
*n*	—	120	60	180
L% recovery first hour	—	0.05 (0.03, 0.10)	0.03 (0.01, 0.06)	0.04 (0.02, 0.08)
L% recovery 2-h cumulative	—	0.14 (0.10, 0.23)	0.09 (0.05, 0.13)	0.12 (0.08, 0.19)
R% recovery first hour	—	0.68 (0.29, 1.13)	0.28 (0.10, 0.71)	0.54 (0.19, 1.01)
R% recovery 2-h cumulative	—	1.99 (1.37, 2.91)	1.04 (0.48, 1.64)	1.66 (0.96, 2.50)
L:R first hour	—	0.08 (0.05, 0.13)	0.10 (0.05, 0.18)	0.08 (0.05, 0.15)
L:R 2-h cumulative	—	0.08 (0.05, 0.12)	0.08 (0.05, 0.16)	0.08 (0.05, 0.13)
L% recovery^3^ and L:R^4^ above reference range, *n* (%)				
Lactulose first hour	—	68 (65.4)	21 (40.4)	89 (57.1)
Lactulose 2-h cumulative	—	103 (98.1)	48 (80.0)	151 (91.5)
L:R first hour	—	23 (22.3)	19 (36.5)	42 (27.1)
L:R 2-h cumulative	—	20 (19.0)	16 (26.7)	36 (21.8)

Abbreviations: BEECH, Biomarkers of Environmental Enteropathy in Children; BEED, Bangladesh Environmental Enteric Dysfunction; CAL, calprotectin; L, lactulose; L:R, lactulose: rhamnose ratio; MPO, myeloperoxidase; NEO, neopterin; R, rhamnose; SEEM, Study of Environmental Enteropathy and Malnutrition.

∗ Values are presented as median (IQR) unless otherwise noted.

1 All stool samples were collected prior to endoscopy. All but 8 (SEEM) urine samples for dual sugar testing were collected prior to endoscopy.

2 Among children with any fecal biomarker data retained in final analyses.

3 Ninty-fifth percentile of L% recovery in 1-h post dose urine collection in United States reference population = 0.033.

4 Ninty-fifth percentile of L:R in 1-h post dose urine collection in United States reference population = 0.147.

The median concentration of CAL was lower in BEECH compared to BEED, and MPO and NEO concentrations were lower in the BEED cohort compared to SEEM; however, ranges overlapped between centers considerably.

Most children passed urine in both the first and second hour postdose intervals: 100 (83%) and 39 (65%) in the BEED and SEEM cohorts, respectively. Of the remainder, 16 (80%) BEED and 8 (38%) SEEM children voided only in the second but not the first collection hour. Median urinary lactulose and rhamnose percent recoveries were greater in the BEED compared to the SEEM cohort in both the first hour, second hour, and 2-h cumulative samples. Median L:R was similar between the 2 cohorts although slightly greater in SEEM samples, driven by the proportionally lower rhamnose excretion in SEEM compared to BEED samples.

Compared with values from a United States reference cohort based on one postdose hour of urine collection [8,12], over half of children in our study (*n* = 89 [57%]) had 1-h L% recovery greater than the reference 95th percentile, although most 2-h cumulative values exceeded this threshold (*n* = 151 [92%]). Twenty-seven percent of first hour and 22% of 2-h cumulative L:R values exceeded the 95th percentile of the reference population.

Table 4 describes univariate regression results for fecal biomarkers; associations with histology significant at the *P* < 0.1 level were further tested in multivariable models adjusted for age, center, and biomarker sample biopsy time interval as displayed in Table 5. Four fecal biomarker–histology pairs were statistically significantly associated (at the *P* < 0.05 level) in multivariable models. Every 1-unit increase in IEL score was associated with a 19% higher mean CAL concentration (exponentiated [exp.] coefficient: 1.19; 95% confidence interval [CI]: 1, 1.41; *P* = 0.044). Intramucosal Brunner’s glands were associated with MPO (exp. coefficient: 1.33; 95% CI: 1.05, 1.69; *P* = 0.018) and NEO (exp. coefficient: 1.37; 95% CI: 1.1, 1.7; *P* = 0.004), and chronic inflammation with NEO (exp. coefficient: 1.61; 95% CI: 1.17, 2.17; *P* = 0.003).

**TABLE 4:** Univariate linear regression results for log fecal calprotectin, myeloperoxidase, and neopterin association with histology scores∗

Histology variables (range of possible scores)	*n*	Calprotectin	*n*	Myeloperoxidase	*n*	Neopterin
		Exp. coefficient (95% CI)		Exp. coefficient (95% CI)		Exp. coefficient (95% CI)
Total score percent-5 (0%–100%)	153	1.01 (0.99, 1.02)	156	1 (0.98, 1.02)	158	0.98 (0.97, 0.99)
Goblet cell depletion (0–4)	180	1.20 (1.0, 1.43)	181	0.96 (0.76, 1.21)	183	1.03 (0.84, 1.28)
Intraepithelial lymphocytes (0–4)	180	1.22 (1.03, 1.43)	180	1.17 (0.93, 1.46)	182	1.05 (0.85, 1.29)
Intramucosal Brunner’s glands (0–3)	177	1.03 (0.85, 1.25)	175	1.35 (1.06, 1.73)	177	1.39 (1.11, 1.73)
Paneth cell depletion (0–3)	112	1.13 (0.93, 1.37)	115	0.82 (0.65, 1.04)	117	0.85 (0.7, 1.02)
Villus architecture (0–4)	127	1.0 (0.87, 1.15)	118	1.04 (0.84, 1.28)	120	0.85 (0.71, 1.01)
Chronic inflammation (0–3)	178	1.06 (0.84, 1.34)	178	1.27 (0.9, 1.8)	180	1.57 (1.15, 2.16)
Enterocyte injury (0–3)	181	1.15 (0.81, 1.62)	181	0.89 (0.53, 1.48)	183	1.03 (0.64, 1.64)
Epithelial detachment (0–4)	182	1.03 (0.84, 1.25)	181	1.22 (0.92, 1.62)	183	1.15 (0.89, 1.5)

Abbreviations: CI, confidence interval; Exp., exponentiated.

∗ Associations significant at the *P* < 0.1 level in the univariate linear regression models (presented in this table) were further considered in multivariable models. 95% CIs are presented for comparability to CIs in multivariable models. The following biomarker–histology pairs were significant at the *P* < 0.1 level: calprotectin–goblet cell depletion, calprotectin–intraepithelial lymphocytes, myeloperoxidase–intramucosal Brunner’s glands, neopterin–total score percent-5, neopterin–intramucosal Brunner’s glands, neopterin–Paneth cell depletion, neopterin–villus architecture, neopterin–chronic inflammation. Exponentiated coefficients can be interpreted as the percent change in the biomarker for each unit increase in the histology score, with an exponentiated coefficient of 1, <1, and >1 meaning no, inverse, and positive relationship between the biomarker and histology score, respectively. For example, a 20% increase in calprotectin concentration was seen with each 1-unit increase in the goblet cell depletion score.

**TABLE 5:** Multivariable linear regression results for log fecal calprotectin, myeloperoxidase, and neopterin association with histology scores∗

Histology variables (range of possible scores)	*n*	Calprotectin	*n*	Myeloperoxidase	*n*	Neopterin
		Exp. coefficient (95% CI)		Exp. coefficient (95% CI)		Exp. coefficient (95% CI)
Total score percent-5 (0%–100%)	—	—	—	—	158	0.99 (0.97, 1.00)
Goblet cell depletion (0–4)	181	1.14 (0.95, 1.38)	—	—	—	—
Intraepithelial lymphocytes (0–4)	181	1.19 (1, 1.41)	—	—	—	—
Intramucosal Brunner’s glands (0–3)	—	—	175	1.33 (1.05, 1.69)	177	1.37 (1.1, 1.7)
Paneth cell depletion (0–3)	—	—	—	—	117	1.01 (0.78, 1.32)
Villus architecture (0–4)	—	—	—	—	120	0.85 (0.72, 1.01)
Chronic inflammation (0–3)	—	—	—	—	180	1.61 (1.17, 2.17)

Abbreviations: CI, confidence interval; Exp., exponentiated.

∗ All associations in univariate linear regression models significant at the *P* < 0.1 level were included in multivariable linear regressions adjusted for age, biomarker sample biopsy time interval, and study site; 95% CIs are presented for both univariate and multivariable models for comparability. Exponentiated coefficients can be interpreted as the percent change in the biomarker for each unit increase in the histology score, holding covariates constant, with an exponentiated coefficient of 1, <1, and >1 meaning no, inverse, and positive relationship between the biomarker and histology score, respectively. For example, a 19% increase in calprotectin concentration was seen with each 1-unit increase in the intraepithelial lymphocyte score, holding covariates constant.

Univariate and multivariable regression results for 2-h cumulative L% recovery and L:R are displayed in TABLE 6, TABLE 7. IELs remained significantly associated with L% recovery in multivariable analysis (1.22; 95% CI: 1.05, 1.41), although the relationship between L:R and IELs and epithelial detachment neared significance. We also conducted a sensitivity analysis using first hour measurements (Supplemental Tables 3 and 4) and observed that L:R was associated with both enterocyte injury and epithelial detachment.

**TABLE 6:** Univariate linear regression results for log 2-h cumulative lactulose percentage recovery and log 2-h cumulative L:R association with histology scores∗

Histology variables (range of possible scores)	*n*	Lactulose % recovery cumulative Exp. coefficient (95% CI)	L:R cumulative Exp. coefficient (95% CI)
Total score percent-5 (0%–100%)	128	1.02 (1.00, 1.03)	1.00 (1.00, 1.01)
Goblet cell depletion (0–4)	165	1.20 (1.02, 1.42)	0.92 (0.80, 1.05)
Intraepithelial lymphocytes (0–4)	164	1.16 (0.99, 1.36)	1.13 (0.99, 1.29)
Intramucosal Brunner’s glands (0–3)	162	0.92 (0.77, 1.09)	1.00 (0.87, 1.15)
Paneth cell depletion (0–3)	113	1.18 (1.00, 1.38)	0.99 (0.87, 1.12)
Villus architecture (0–4)	116	1.04 (0.90, 1.20)	1.05 (0.94, 1.19)
Chronic inflammation (0–3)	163	0.91 (0.71, 1.16)	0.95 (0.78, 1.17)
Enterocyte injury (0–3)	165	0.98 (0.68, 1.41)	1.33 (0.99, 1.79)
Epithelial detachment (0–4)	165	1.01 (0.83, 1.23)	1.13 (0.96, 1.33)

Abbreviations: CI, confidence interval; Exp., exponentiated; L:R, lactulose-to-rhamnose ratio.

∗ Associations significant at the *P* < 0.1 level in the univariate linear regression models (presented in this table) were further considered in multivariable models. 95% CIs are presented for comparability to CIs in multivariable models. The following biomarker–histology pairs were significant at the *P* < 0.1 level: lactulose % recovery–total score percent-5, lactulose % recovery–goblet cell depletion, lactulose % recovery–intraepithelial lymphocytes, lactulose % recovery–Paneth cell depletion, L:R–intraepithelial lymphocytes, L:R–enterocyte injury. Exponentiated coefficients can be interpreted as the percent change in the lactulose % recovery or L:R for each unit increase in the histology score, with an exponentiated coefficient of 1, <1, and >1 meaning no, inverse, and positive relationship between the biomarker and histology score, respectively. For example, a 2% increase in lactulose % recovery was seen with each 1-unit increase in total score percent-5.

**TABLE 7:** Multivariable linear regression results for log 2-h cumulative lactulose % recovery and log 2-h cumulative L:R association with histology scores

Histology variables (range of possible scores)	*n*	Lactulose % recovery cumulative Exp. coefficient (95% CI)	L:R cumulative Exp. coefficient (95% CI)
Total score percent-5 (0%–100%)	128	1.00 (0.99, 1.01)	—
Goblet cell depletion (0–4)	165	1.00 (0.84, 1.19)	—
Paneth cell depletion (0–3)	113	0.83 (0.67, 1.02)	—
Intraepithelial lymphocytes (0–4)	164	1.22 (1.05, 1.41)	1.12 (0.98, 1.28)
Enterocyte injury (0–3)	165	—	1.32 (0.98, 1.77)

All associations in univariate linear regression models with p<0.1 were further explored in multivariable linear regressions and adjusted for age, biomarker sample biopsy time interval, and study site. Exponentiated coefficients can be interpreted as the percent change in the lactulose % recovery or L:R for each unit increase in the histology score, holding covariates constant with an exponentiated coefficient of 1, <1, and >1 meaning no, inverse, and positive relationship between the biomarker and histology score, respectively. For example, a 12% increase in L:R was seen with each one-unit increase in intraepithelial lymphocytes, holding covariates constant; although this finding did not reach statistical significance.

Abbreviations: CI, confidence interval; Exp., exponentiated; L:R, lactulose-to-rhamnose ratio.

**FIGURE 2:**
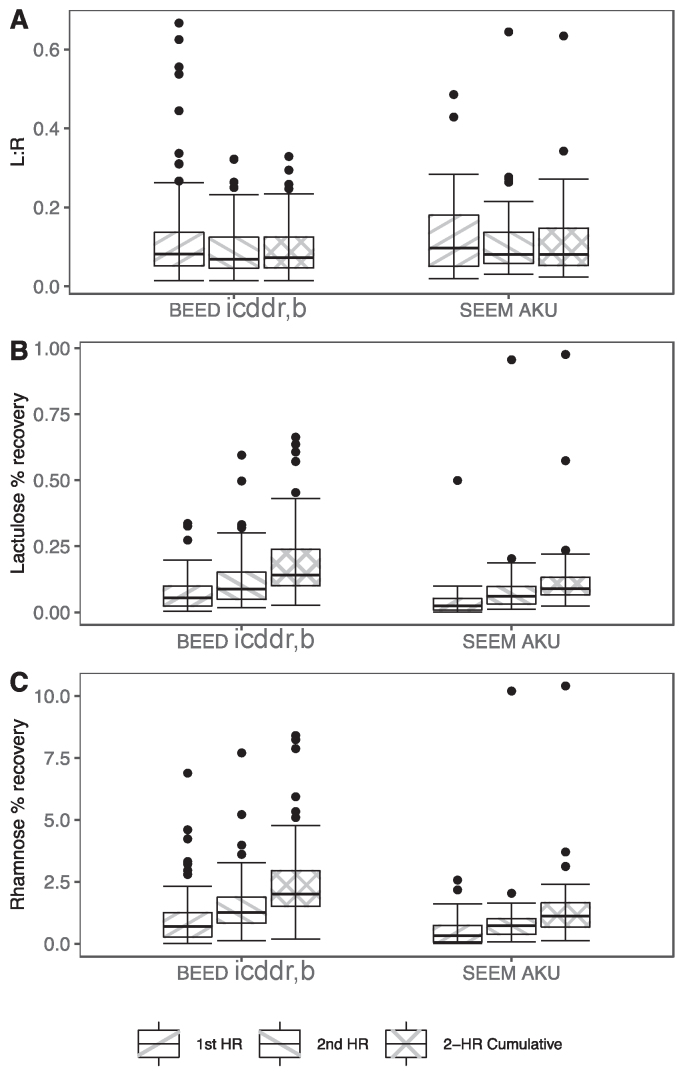
First hour, second hour, and 2-h cumulative L:R (A), lactulose percentage recovery (B), and rhamnose percentage recovery (C) among participants who voided in both the first and second hour. AKU, Aga Khan University; BEED, Bangladesh Environmental Enteric Dysfunction; icddr,b, International Centre for Diarrhoeal Disease Research, Bangladesh; L:R, lactulose-to-rhamnose ratio; SEEM, Study of Environmental Enteropathy and Malnutrition.

Lastly, we explored how first hour, second hour, and 2-h cumulative sugar excretions and L:R differed among children who voided in both the first and second hour collection intervals (Figure 2). The median L% and rhamnose percentage recoveries were 2-fold greater in the second hour than the first hour collection (both centers combined, data not shown), and the 2-h cumulative sugar recovery results more closely resembled those from the second hour collection. L:R was similar across the time intervals, although slightly greater in the first hour than second hour and 2-h cumulative because of the relatively lower excretion of rhamnose compared to lactulose in the first compared with second hour. Supplemental Figure 1 describes individual participant-level L:R and L% and rhamnose percentage recovery results and shows that 2-h cumulative and second hour measurements were fairly close, while first hour values often differed, some quite substantially.

## Discussion

We found several associations between histologic features and biomarkers of host response in children with EED. First, fecal NEO, a marker of Th1 immune activation, was the only marker associated with the EED histology index score (the TSP-5) and with duodenal chronic inflammation. The latter was the strongest histology–biomarker association in our study. Fecal NEO concentrations have been associated with EED and growth faltering [[13], [14], [15]], although in one Brazilian study, the association depended on simultaneously elevated fecal MPO concentrations [16]. Although chronic inflammation has been noted in EED [[17], [18], [19], [20], [21]] and was common in tissue from children with EED in this study, it was also present in tissue from children with celiac disease or no identifiable gastrointestinal disorder or clinical histopathologic diagnosis [7]. Nonetheless, chronic inflammation and fecal NEO, though not specific to EED, may still represent a treatment target and a surrogate marker for this disorder, respectively.

Some fecal marker–histology relationships were less anticipated. Fecal CAL concentrations, which have been used as a biomarker in EED studies [22], were associated with small bowel IELs, a hallmark of EED [7,23,24]. Fecal CAL is used clinically to distinguish inflammatory bowel disease from functional disorders and to monitor disease progression. Fecal CAL indirectly measures neutrophil migration into intestinal mucosa [23,25,26], but as described elsewhere in this issue, neutrophils were rare in EED small bowel biopsies [7]. Possible explanations for the observed association include that IELs synthesize CAL or IELs reflect more distal neutrophil-mediated gut injury in EED.

Fecal MPO and NEO associations with intramucosal Brunner’s glands were also unexpected. Brunner’s glands consist of nonphagocytic cells and are normally found in the mucosa of the stomach and first segment of the duodenum. They secrete mucus-rich alkaline fluid that protects the proximal duodenum from acidic chyme [27,28]. NEO is produced by macrophages, and MPO reflects polymorphonuclear leukocyte activity. Neither NEO nor MPO is known to be produced by Brunner’s glands; however, intestinal inflammation has been observed in rare cases of intramucosal Brunner’s gland hyperplasia and duodenal hamartoma [29]. In summary, we recommend that fecal MPO, NEO, and CAL continue to be used in research settings; their value as markers of EED will emerge or be refuted.

Our data also illuminate permeability tracer uptake kinetics. The dual sugar test is based on the premise that the ingested monosaccharide (mannitol or rhamnose) traverses both permeable and intact gut barriers, and therefore enters the circulation and is recovered in the urine in disease and health. In contrast, the larger sugar, lactulose, minimally traverses intact mucosal barriers and is only excreted in urine to any substantial extent if the gut mucosa is damaged. Gut permeability is measured by postoral administration elevated L% recovery or disaccharide to monosaccharide ratios [[30], [31], [32]]. Urinary lactulose mannitol ratios (L:M) exceeded United Kingdom reference values in Asian, African, and South American children who were asymptomatic, undernourished, or had diarrhea [[33], [34], [35], [36], [37]]. L:M was inversely associated with LAZ in Gambian children [14,34].

Dual sugar tests traditionally assay urine collected for 3–5 h after ingestion [31], but shorter intervals have been proposed when high sensitivity mass spectrometry is used in conjunction with liquid chromatography [8]. Our study enabled us to compare postingestion 1-h to 2-h urinary sugar recovery. First, we found substantially more recovery of both sugars in the second than the first hour postdose, and 2-h cumulative values closely aligned with second hour recoveries. Differences in prediction of histopathology also emerged. Specifically, at 1 h, the L:R (but not L% recovery) was associated with enterocyte injury and epithelial detachment, lesions we predesignated as histologic features most closely related to a disrupted barrier. Interestingly, in children with treatment-naïve celiac disease, 1-h urinary L:R, but not L% recovery, correlated with the Marsh histology score [38]. However, in our study, after 2 cumulative hours of collection, L% recovery became significantly associated with duodenal IELs, while the L:R lost association with any histologic finding.

As far as we are aware, this is the first study to demonstrate an association between the specific inflammatory effector cells, IELs, and lactulose recovery. However, Campbell et al. [23] described an association between IELs and L:M (L% recovery was not reported) among Gambian children. Although they also identified many cytokines of lymphocytic origin by tissue staining, they did not report whether they sought associations between cytokines and L:M. Some cytokines released from IEL-derived T cells maintain gut barrier homeostasis (e.g., IL-2, IL-4) while others (e.g., interferon-γ, tumor necrosis factor) lead to barrier disruption [[39], [40], [41]]. Cytokine-mediated effects on intestinal integrity have been described in celiac disease, a disorder in which IELs are also a predominant feature [42,43]. The only other dual sugar-histology investigations that we are aware of identified inverse relationships between L:R with villus height and villus perimeter in Zambian children with severe acute malnutrition and histologic features of EED [44]. Although we did not identify a relationship between L:R or L% recovery with our semiquantitative villus architecture score, we did observe L:R (at 1 h) associations with epithelial detachment and enterocyte injury, which are intuitively histologic correlates of increased permeability. It is unclear why we see associations with 1-h but not 2-h cumulative L:R. Nonetheless, our data endorse urine collections of at least 2 h when performing dual sugar testing, because of substantially greater recovery of probes in hour 2.

We acknowledge we are unable to account for renal clearance, hydration status, and predose sugar exposures. Also, the 10-min difference in postdose urine collection start times between SEEM and BEED may have contributed to variance in sugar contact with the small bowel barrier. However, these factors are unlikely to explain differences in associations with histology between the timed voids. It is also likely that collection over 2 h smooths out interindividual variability related to upper gastrointestinal motility differences. Intestinal inflammation has been suggested to be in the causal pathway leading to barrier dysfunction [2], and our finding of a 22% higher L% recovery with each 1-unit increase in the IEL density score, a large effect size, supports this concept.

Immunohistochemistry and transcriptomics reported elsewhere in this issue suggest lipocalin 2 and regenerating gene 1β protein as important EED tissue markers [45,46]. Their fecal concentrations should be studied in future EED histology studies, especially in view of data suggesting their utility in enteropathy and stunting [[47], [48], [49]]. Our testing for multiple associations increases the possibility that one or more associations were due to chance. Additional limitations include variability in intervals between samples for biomarkers and biopsy, which reflect intercenter protocol differences that could not be corrected after the fact [6]. However, we adjusted for center in analyses, and it is doubtful that the degree of histologic abnormalities observed developed in the interval between specimen collection and biopsy. Hence, it is likely that the biomarkers we studied were present well before the endoscopies, though longer intervals for some participants might have attenuated our ability to identify some associations. Although we present multicenter data, differences in protocols and biomarkers assessed may limit the generalizability of our findings to other EED endemic settings.

In summary, these unique studies with overarching similarities are the first pediatric EED biopsy studies in the modern era and the first systematic assessment of EED biomarker–histology relationships. In many mucosal gastrointestinal illnesses of childhood, such as inflammatory bowel disease and eosinophilic esophagitis, biomarkers have yet to supplant the need for mucosal assessment and reassessments. However, cost, expertise required, and risk considerations limit endoscopy performance to children who stand to gain clinical benefit, especially in low- and middle-income countries [50]. Nonetheless, our ability to link histology data generated by a consensus of expert pathologists using a standardized and validated scoring system and 4 biomarkers commonly used in EED research is encouraging for continued work in the field. This novel multicountry approach justifies further EED biomarker discovery and validation.

## Figures and Tables

**Table:** 

Surname	Name	Full Affiliation	Email
Ahmed	Kumail	Department of Paediatrics and Child Health, Aga Khan University, Karachi, Pakistan	kumail.ahmed@aku.edu
Ahmed	Sheraz	Department of Paediatrics and Child Health, Aga Khan University, Karachi, Pakistan	sheraz.ahmed@aku.edu
Ahmed	Tahmeed	Nutrition Research Division, International Centre for Diarrhoeal Disease Research, Bangladesh, Dhaka, Bangladesh	tahmeed@icddrb.org
Alam	Md Ashraful	Nutrition Research Division, International Centre for Diarrhoeal Disease Research, Bangladesh, Dhaka, Bangladesh	mashraful@icddrb.org
Amadi	Beatrice	Tropical Gastroenterology and Nutrition Group, University of Zambia School of Medicine, Lusaka, Zambia	beatriceamadi@ymail.com
Begum	SM Khodeza Nahar	Department of Pathology, Bangladesh Specialized Hospital, Dhaka, Bangladesh	khodeza33@hotmail.com
Besa	Ellen	Tropical Gastroenterology & Nutrition Group, University of Zambia School of Medicine, Lusaka, Zambia	ellen@tropgan.net
Fahim	Shah Mohammad	Nutrition Research Division, International Centre for Diarrhoeal Disease Research, Bangladesh, Dhaka, Bangladesh	mohammad.fahim@icddrb.org
Gazi	Md Amran	Nutrition Research Division, International Centre for Diarrhoeal Disease Research, Bangladesh, Dhaka, Bangladesh	amran.gazi@icddrb.org
Gilchrist	Carol A	Department of Medicine, University of Virginia, Charlottesville, Virginia School of Medicine, USA	cg2p@virginia.edu
Haque	Rashidul	Infectious Disease Division, International Centre for Diarrhoeal Disease Research, Bangladesh, Dhaka, Bangladesh	rhaque@icddrb.org
Hasan	Md Mehedi	Nutrition Research Division, International Centre for Diarrhoeal Disease Research, Bangladesh, Dhaka, Bangladesh	md.hasan@icddrb.org
Hossain	Md Shabab	Nutrition Research Division, International Centre for Diarrhoeal Disease Research, Bangladesh, Dhaka, Bangladesh	dr.shabab@icddrb.org
Hotwani	Aneeta	Department of Paediatrics and Child Health, Aga Khan University, Karachi, Pakistan	aneeta.hotwani@aku.edu
Hussain	Shahneel	Department of Biological and Biomedical Sciences, Aga Khan University, Karachi, Pakistan	shahneel.hussain@aku.edu
Iqbal	Junaid	Department of Paediatrics and Child Health, Aga Khan University, Karachi, Pakistan	junaid.iqbal@aku.edu
Jakhro	Sadaf	Department of Paediatrics and Child Health, Aga Khan University, Karachi, Pakistan	sadaf.jakhro@aku.edu
Kabir	Furqan	Department of Paediatrics and Child Health, Aga Khan University, Karachi, Pakistan	furqan.kabir@aku.edu
Liu	Ta-Chiang	Department of Pathology and Immunology, Washington University School of Medicine, St. Louis, MO, USA	ta-chiang.liu@wustl.edu
Mann	Barbara J	Department of Medicine, University of Virginia School of Medicine, Charlottesville, Virginia, USA	bjm2r@virginia.edu
Mazumder	Ramendra Nath	Nutrition Research Division, International Centre for Diarrhoeal Disease Research, Bangladesh, Dhaka, Bangladesh	ramen@icddrb.org
Memon	Waheeda	Department of Paediatrics and Child Health, Aga Khan University, Karachi, Pakistan	waheeda.memon@aku.edu
Moskaluk	Christopher A	Department of Pathology, University of Virginia School of Medicine, Charlottesville, VA, USA	cam5p@virginia.edu
Qureshi	Abdul Khalique	Department of Paediatrics and Child Health, Aga Khan University, Karachi, Pakistan	abdul.qureshi@aku.edu
Ragahavan	Shyam S	Department of Pathology, University of Virginia School of Medicine, Charlottesville, VA, USA	SR7WQ@hscmail.mcc.virginia.edu
Rahman	Masudur	Department of Gastroenterology, Sheikh Russel National Gastroliver Institute and Hospital, Dhaka, Bangladesh	drmasud47@yahoo.com
Rahman	Najeeb	Department of Paediatrics and Child Health, Aga Khan University, Karachi, Pakistan	najeeb.rahman@aku.edu
Sadiq	Kamran	Department of Paediatrics and Child Health, Aga Khan University, Karachi, Pakistan	kamran.sadiq@aku.edu
Sarker	Shafiqul Alam	Nutrition Research Division, International Centre for Diarrhoeal Disease Research, Bangladesh, Dhaka, Bangladesh	sasarker@icddrb.org
Sullivan	Peter B	Department of Paediatrics, Children's Hospital, University of Oxford, Oxford, UK	peter.sullivan@paediatrics.ox.ac.uk
Tearney	Guillermo J	Department of Pathology, Harvard Medical School, Boston, MA, USA	gtearney@partners.org
Umrani	Fayaz	Department of Paediatrics and Child Health, Aga Khan University, Karachi, Pakistan	fayaz.umrani@aku.edu
Yilmaz	Omer H	Department of Pathology, Massachusetts General Hospital, Boston, MA, USA	oyilmaz@mgh.harvard.edu
Zyambo	Kanekwa	Tropical Gastroenterology & Nutrition Group, University of Zambia School of Medicine, Lusaka, Zambia	kanekwa@tropgan.net

## Data Availability

Data described in the manuscript, code book, and analytic code will be made available upon request to the corresponding author pending application and approval.
