# Supplementary material for: Biomarker relationships with small bowel histopathology among malnourished children with environmental enteric dysfunction in a multicountry cohort study
Source: Am J Clin Nutr. 2024 Sep 17;120(Suppl 1):S73–83. doi: 10.1016/j.ajcnut.2024.02.029 (PMC13169017; doi:10.1016/j.ajcnut.2024.02.029)
Supplement: Multimedia component 1 [file mmc1.zip › ajcnut_471_MUSTAF~1_mmc1.DOC]

Supplemental Appendix

Power Calculations

We calculated Cohen’s ƒ^2^ statistics for each biomarker to determine detectable effect sizes based on an alpha level of 0.1, power of 0.8 and the largest sample size for each biomarker using the ‘pwr’ package in R. Because the fecal biomarker sample sizes are very similar (CAL=182, MPO=181, NEO=183) we used the middle sample size here (n=182) for all fecal biomarkers. The sample size for L:R is 165. We find ƒ^2^ = 0.034 for fecal biomarkers and ƒ^2^ = 0.037 for L:R. For reference an ƒ^2^ of 0.02 generally represents a small effect size while an ƒ^2^ of 0.15 represents a medium effect size.

Supplemental Table 1a. Pre-dose urine measurements among samples included in LR analysis. Among children with more than one dual sugar test, only pre-dose measurements from the LR test conducted closest to the time of endoscopy are included in this table and in the main LR analysis.

|  | BEED | SEEM | Combined |
| --- | --- | --- | --- |
| Number with LR testing | 120 | 60 | 180 |
| Number (%) pre-dose LR results | 7 (6%) | 9 (15%) | 16 (9%) |
| Number (%) with rhamnose >3x lower limit of detection (LLD) | 2 (29%) | 0 (0%) | 2 (13%) |
| Number (%) with rhamnose >10 ug/mL | 0 (0%) | 0 (0%) | 0 (0%) |
| Number (%) with lactulose >3x LLD | 2 (29%) | 0 (0%) | 2 (13%) |
| Number (%) with lactulose >10 ug/mL | 1 (14%) | 0 (0%) | 1 (6%) |

Supplemental Table 1b. Pre-dose urine measurements among all BEED and SEEM LR tests. Includes all pre-dose LR measurements collected during BEED and SEEM studies. These may include multiple tests per child at different times as well as children who were not eligible for endoscopy.

|  | BEED | SEEM | Combined |
| --- | --- | --- | --- |
| Number of LR tests | 1907 | 313 | 2,200 |
| Number (%) with pre-dose LR results | 20 (1%) | 49 (16%) | 69 (3%) |
| Number (%) with rhamnose >3x lower limit of detection (LLD) | 6 (30%) | 8 (16%) | 14 (20%) |
| Number (%) with rhamnose >10 ug/mL | 0 (0%) | 0 (0%) | 0 (0%) |
| Number (%) with lactulose >3x LLD | 6 (30%) | 10 (20%) | 16 (23%) |
| Number (%) with lactulose >10 ug/mL | 1 (5%) | 2 (4%) | 3 (4%) |

Note: Supplemental Table 1a shows pre-dose LR data for LR samples included in our analysis. Because relatively few of the children included in our analysis had pre-dose LR assessments and to further assess the scope of pre-dose exposure to these sugars in the settings where the studies were conducted, we also include pre-dose data for the entire BEED and SEEM cohorts (Supplemental Table 1b), which may include multiple tests per child based on multiple visits and children who were not eligible for endoscopy due to anthropometric recovery after nutritional intervention.

Based on thresholds defined by Khosbin et al. (1) contamination is considered “significant” if >3x the lower level of assay detection (LLD) and “major” (i.e., potentially interfering with interpretation of post-dose results) if >10 ug/ml. 3x LLD for lactulose in the assay used for this study is 0.3 ug/ml and for rhamnose 0.375 ug/ml. While “significant” contamination was noted with both sugars, “major” contamination was not identified for rhamnose and rare for lactulose. Data on repeat LR testing within two days was not systematically recorded, so we were unable to assess whether repeat testing was related to pre-dose contamination.

1. Khoshbin K, Khanna L, Maselli D, Atieh J, Breen-Lyles M, Arndt K, Rhoten D, Dyer RB, Singh RJ, Nayar S, Bjerkness S, Harmsen WS, Busciglio I, Camilleri M. Development and Validation of Test for "Leaky Gut" Small Intestinal and Colonic Permeability Using Sugars in Healthy Adults. Gastroenterology. 2021 Aug;161(2):463-475.e13. doi: 10.1053/j.gastro.2021.04.020. Epub 2021 Apr 16. PMID: 33865841; PMCID: PMC8328885.

Supplemental Table 2. Description of histology parameter scoring.

| Goblet cell density depletion^1^ | 0: Normal goblet cell density (at least 1 goblet cell per 20 enterocytes) in all evaluable mucosal epithelial layer | 1: Decreased goblet cells (<1/20 enterocytes) in 1-25% of evaluable mucosal epithelium | 2: Decreased goblet cells (<1/20 enterocytes) in 26-50% of evaluable mucosal epithelium | 3: Decreased goblet cells (<1/20 enterocytes) in 51-75% of evaluable mucosal epithelium | 4: Decreased goblet cells (<1/20 enterocytes) in 76-100% of evaluable mucosal epithelium | NS: Not scorable |
| --- | --- | --- | --- | --- | --- | --- |
| Intra  epithelial lymphocytes^1^ | 0: No areas observed with epithelial/lymphocyte ratio >20% | 1: Lymphocyte/ epithelial ratio >20%, but <50%, in less than 50% of mucosa | 2: Lymphocyte/ epithelial ratio >20%, but <50%, in greater than 50% of mucosa | 3: Lymphocyte/ epithelial ratio >50% in less than 50% of mucosa | 4: Lymphocyte/ epithelial ratio >50% in greater than 50% of mucosa | NS: Not scorable |
| Intramucosal Brunner’s glands^1,2^ | 0: None observed | 1: One or two foci of intramucosal Brunner glands, none involving more than 5 crypt bases | 2: 3-5 foci of intramucosal Brunner glands, none involving more than 5 crypt bases | 3: > 5 foci, or any area of intramucosal Brunner glands involving >5 crypt bases |  | NS: Not scorable |
| Paneth cell density depletion^1^ | 0: >5 Paneth cells/ crypt base, on average | 1: 2-4 Paneth cells/ crypt base, on average | 2: <2 Paneth cell/crypt base, involving <50% of crypt bases | 3: <2 Paneth cell/crypt, involving >50% of crypt bases |  | NS: Not scorable |
| Villus architecture^1^ | 0: Majority of villi are >3 crypt lengths long | 1: Villi are < 3 but > 1 crypt lengths long, with abnormality involving ≤ 50% of mucosa | 2: Villi are < 3 but > 1 crypt lengths long, with abnormality involving > 50% of mucosa | 3: Villi absent, or <1 crypt length long, with abnormality involving ≤ 50% of mucosa | 4: Villi absent, or <1 crypt length long, with abnormality involving > 50% of mucosa | NS: Not scorable |
| Chronic Inflam-mation | 0: No qualitative increase in mononuclear inflammatory cells (MIC) in lamina propria. Majority of villus bases contain <3 MIC across, on average | 1: Increased MIC, based on villus base displaying 3-5 MIC across, on average | 2: Increased MIC, based on villus base displaying 6-10 MIC across, on average | 3: Increased MIC, based on villus base displaying >10 lymphocytes on average |  | NS: Not scorable |
| Enterocyte injury | 0: Majority of enterocytes (90%) show tall columnar morphology | 1: Enterocytes show low columnar (<2:1 L:W ratio), cuboidal or flat morphology, in ≤ 50% of mucosa | 2: Enterocytes show low columnar (<2:1 L:W ratio), cuboidal or flat morphology, in > 50% of mucosa | 3: Any area of mucosal erosion/ulceration |  | NS: Not scorable factor |
| Epithelial detachment | 0: Complete coverage of mucosal surface by epithelial cells | 1: Surface epithelium missing or detached from <25% of mucosa | 2: Surface epithelium missing or detached from 25-50% of mucosa | 3: Surface epithelium missing or detached from 51-75% of mucosa | 4: Surface epithelium missing or detached from >75% of mucosa | NS: Not scorable |

**^1^**These five histology parameters are included in the calculation of the Total Score Percent-5 (TSP-5). At least four of these five parameters must have a numeric score (i.e., not non-scorable) for a slide to have a TSP-5 calculated. The numerator of the TSP-5 is the sum of the score of the scorable parameters and the denominator is the sum of the possible maximum score of the scorable parameters.

Abbreviations: NS, not scorable; variable cannot be determined because of slide quality or other factor

Supplemental Table 3: Univariate linear regression associations between log 1-hour lactulose % recovery and log 1-hour L:R with histology scores.

| Histology variables (range of possible scores) |  | 1-hour lactulose % recovery | | 1-hour L:R | |  |
| --- | --- | --- | --- | --- | --- | --- |
|  | N | Exp. Coefficient (95% CI) | N | Exp. Coefficient (95% CI) |  | |
| Total Score Percent-5 (0-100%) | 119 | 1.01 (1.00, 1.03) | 118 | 1.01 (1.00, 1.02) |  | |
| Goblet Cell Depletion (0-4) | 156 | 1.26 (1.01, 1.57) | 155 | 0.89 (0.76, 1.03) |  | |
| Intraepithelial Lymphocytes (0-4) | 155 | 1.07 (0.85, 1.33) | 154 | 1.05 (0.90, 1.22) |  | |
| Intramucosal Brunner’s Glands (0-3) | 153 | 0.94 (0.74, 1.19) | 152 | 1.02 (0.87, 1.20) |  | |
| Paneth Cell Depletion (0-3) | 104 | 1.12 (0.91, 1.38) | 104 | 0.97 (0.85, 1.11) |  | |
| Villus Architecture (0-4) | 107 | 1.00 (0.83, 1.21) | 106 | 1.09 (0.97, 1.23) |  | |
| Chronic Inflammation (0-3) | 154 | 0.85 (0.60, 1.19) | 153 | 0.88 (0.70, 1.11) |  | |
| Enterocyte Injury (0-3) | 156 | 0.64 (0.39, 1.07) | 155 | 1.52 (1.08, 2.15) |  | |
| Epithelial detachment (0-4) | 156 | 0.90 (0.69, 1.18) | 155 | 1.23 (1.03, 1.48) |  | |

Associations significant at the p<0.1 level in the univariate linear regression models (presented in this table) were further considered in multivariable models. 95% CIs are presented for comparability to CIs in multivariable models. The following biomarker-histology pairs were significant at the p<0.1 level: lactulose % recovery-goblet cell depletion, lactulose % recovery-enterocyte injury, LR-enterocyte injury, L:R-epithelial detachment. Exponentiated coefficients can be interpreted as the percent change in the lactulose % recovery or L:R for each unit increase in the histology score, with an exponentiated coefficient of 1, <1, and >1 meaning no, inverse, and positive relationship between the biomarker and histology score, respectively.

Abbreviations: CI, confidence interval; Exp, exponentiated; L:R, lactulose:rhamnose ratio

Supplemental Table 4: Multivariable linear regression associations between log 1-hour lactulose % recovery and log 1-hour LR with histology measurements

| Histology variables  (range of possible scores) |  | 1-hour lactulose % recovery | | 1-hour LR |  |
| --- | --- | --- | --- | --- | --- |
|  | N | Exp. Coefficient (95% CI) | N | Exp. Coefficient (95% CI) | |
| Goblet Cell Depletion (0-4) | 156 | 1.03 (0.81, 1.32) |  |  | |
| Enterocyte Injury (0-3) | 156 | 0.69 (0.42, 1.12) | 155 | 1.50 (1.06, 2.12) | |
| Epithelial detachment (0-4) |  |  | 155 | 1.22 (1.02, 1.47) | |

All associations in univariate linear regression models with p<0.1 were further explored in multivariable linear regressions and adjusted for age, biomarker sample-biopsy time interval, and study site. Exponentiated coefficients can be interpreted as the percent change in the lactulose % recovery or L:R for each unit increase in the histology score, holding covariates constant with an exponentiated coefficient of 1, <1, and >1 meaning no, inverse, and positive relationship between the biomarker and histology score, respectively.

Abbreviations: CI, confidence interval; Exp, exponentiated; LR, lactulose:rhamnose ratio

Supplemental Figure 1. Scatter plots showing the distribution of 1st hour, 2nd hour, and 2-hr cumulative L:R (A), lactulose % recovery (B), and rhamnose% recovery (C) measurements by site.

Each row represents one individual and 1^st^ hour and 2^nd^ hour measurements per individual are connected by a horizontal line. Each panel is arranged in descending order of cumulative L:R measurements.
