# Supplementary material for: Biomarker relationships with small bowel histopathology among malnourished children with environmental enteric dysfunction in a multicountry cohort study
Source: Am J Clin Nutr. 2024 Sep 17;120(Suppl 1):S73–83. doi: 10.1016/j.ajcnut.2024.02.029 (PMC13169017; doi:10.1016/j.ajcnut.2024.02.029)

Biomarker relationships with small bowel histopathology among malnourished children with environmental enteric dysfunction in a multi-country cohort study Mahfuz, Mustafa

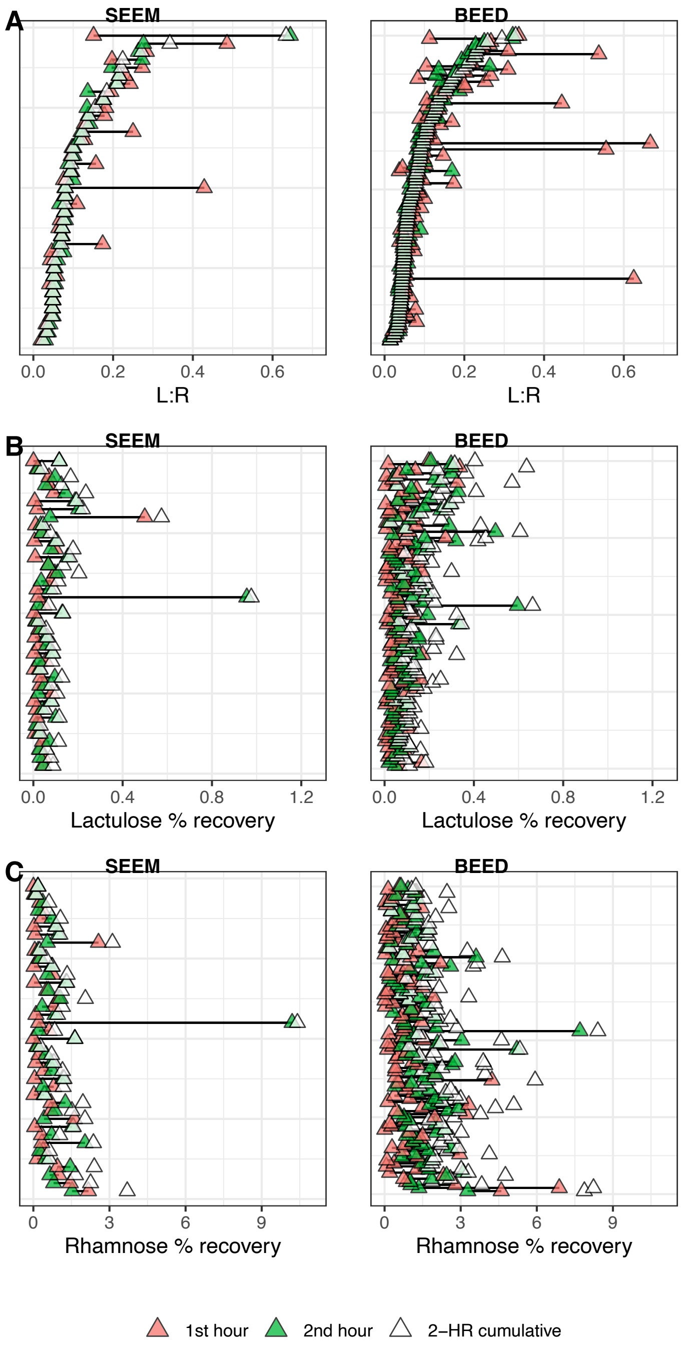

Supplement: Multimedia component 2 [file mmc2.zip › ajcnut_471_MUSTAF~2_mmc2.PDF]
